# Real-world treatment patterns and outcomes of abemaciclib for the treatment of HR + , HER2- metastatic breast cancer patients in Japan

**DOI:** 10.1007/s12282-023-01461-6

**Published:** 2023-05-22

**Authors:** K. Nozawa, M. Terada, M. Onishi, Y. Ozaki, T. Takano, W. Fakhouri, D. Novick, J. M. Haro, L. H. Faris, T. Kawaguchi, Y. Tanizawa, Junji Tsurutani

**Affiliations:** 1 Department of Breast Oncology, Aichi Cancer Center Hospital, Nagoya, Japan; 2 Department of Breast Surgery, Graduate School of Medical Sciences, Nagoya City University, Nagoya, Japan; 3 Department of Medical Oncology, National Cancer Center Hospital, Tokyo, Japan; 4 Department of Breast Medical Oncology, Breast Oncology Center, The Cancer Institute Hospital of the Japanese Foundation for Cancer Research, Tokyo, Japan; 5 Eli Lilly and Company, Indianapolis, IN USA; 6 Parc Sanitari Sant Joan de Déu, Universitat de Barcelona, Sant Boi de Llobregat, Barcelona, Spain; 7 Advanced Cancer Translational Research Institute, Showa University, Tokyo, Japan

**Keywords:** Advanced breast cancer, Estrogen receptor, Progesteron receptor, HER2-negative, Cyclin-dependent kinas

## Abstract

**Introduction:**

This study described, in routine clinical practice in Japan, the patient characteristics, treatment patterns, and outcomes of female patients with HR + /HER2- metastatic breast cancer (MBC) who started abemaciclib treatment.

**Methods:**

Clinical charts were reviewed for patients starting abemaciclib in 12/2018–08/2021 with a minimum of 3 months follow-up data post-abemaciclib initiation regardless of abemaciclib discontinuation. Patient characteristics, treatment patterns, and tumor response were descriptively summarized. Kaplan–Meier curves estimated progression-free survival (PFS).

**Results:**

200 patients from 14 institutions were included. At abemaciclib initiation, median age was 59 years, and the Eastern Cooperative Oncology Group performance status score was 0/1/2 for 102/68/5 patients (58.3/38.9/2.9%), respectively. Most had an abemaciclib starting dose of 150 mg (92.5%). The percentage of patients receiving abemaciclib as 1st, 2nd, or 3rd line treatment was 31.5%, 25.8%, and 25.2%, respectively. The most frequent endocrine therapy drugs used with abemaciclib were fulvestrant (59%) and aromatase inhibitors (40%). Evaluation of tumor response was available for 171 patients, 30.4% of whom had complete/partial response. Median PFS was 13.0 months (95% CI 10.1–15.8 months).

**Conclusions:**

In a routine clinical practice setting in Japan, patients with HR + , HER2- MBC appear to benefit from abemaciclib treatment in terms of treatment response and median PFS, with the results broadly reflecting the evidence demonstrated in clinical trials.

**Supplementary Information:**

The online version contains supplementary material available at 10.1007/s12282-023-01461-6.

## Introduction

Breast cancer is the most commonly diagnosed cancer in women with 2.3 million women diagnosed with the disease in 2020 [1]. In recent decades, significant advances in the treatment options available for patients diagnosed with breast cancer, together with earlier detection arising from organized screening have seen a decline in mortality across the developed world [2]. Nonetheless, breast cancer mortality is significant with more women dying of breast cancer than any other site [1]. In Japan, the incidence of breast cancer is rising, especially so in post-menopausal women [3, 4] and it is projected that over 94,000 women will be diagnosed with breast cancer in Japan in 2022 [5].

While most patients receiving a breast cancer diagnosis present with earlier stage disease [6], approximately 6–10% of patients are diagnosed with advanced or metastatic breast cancer (MBC) and 20–30% of patients diagnosed with earlier-stage disease develop MBC in time [7]. There is no cure for MBC but gains in survival have been observed [8–10].

Evidence has emerged from several randomized controlled trials (RCT) to indicate that inhibitors for cyclin-dependent kinase 4 (CDK4) and 6 (CDK6), namely CDK4/6 inhibitors, delivered alongside established endocrine therapy (ET) regimens are effective in patients with hormone receptor (HR)-positive, human epidermal growth factor 2 receptor (HER2)-negative disease [11]. They now represent the standard of care for patients with this diagnosis. Abemaciclib is a selective small molecule inhibitor of CDK4 and CDK6 administered orally on a continuous twice-daily dosing regimen [12–14]. In preclinical investigation, continuous inhibition of CDK4 and CDK6 by abemaciclib led to cell cycle arrest and death of cancer cells [13, 15]. Based on international trial evidence (MONARCH 2 and MONARCH 3) [16–18], abemaciclib has been approved and available in Japan for the treatment of HR + , HER2- MBC since November 2018. Recent Japan-specific subpopulation studies of these trials have demonstrated the consistent trend with the overall population both regarding effectiveness and safety profile [19–21].

In this study, we aimed to describe the clinicopathological characteristics, treatment patterns, and treatment sequences of patients who were treated with abemaciclib for HR + /HER2- metastatic breast cancer in real-world clinical practice in Japan and assess if abemaciclib was being prescribed in accordance with the relevant guidance. We also aimed to assess treatment effectiveness with treatment response and progression-free survival of those patients.

## Methods

### Study participants and data collection methods

The data for this study was drawn from a clinical chart review of Japanese women either diagnosed initially with, or progressed to, HR + HER2- MBC and who had initiated treatment with abemaciclib between December 2018 and August 2021 in routine clinical practice. To be eligible, patients needed to be adult females (> 18 years) with a confirmed diagnosis of HR + /HER2- metastatic breast cancer (stage IIIb, IIIc or IV), as defined in the relevant American Society of Clinical Oncology (ASCO)/College of American Pathologists (CAP) guidelines [22, 23]. They also needed at least 3 months of follow-up data post abemaciclib initiation (regardless of abemaciclib discontinuation). Patients who were enrolled in another clinical trial any time after the initiation of abemaciclib were excluded. Target sample size was 200 patients. Given that this is a descriptive study, sample size was calculated based on the desired precision of the estimates. The review was conducted over 14 sites by physicians. These sites were mostly specialized cancer centers or university teaching hospitals and were spread across Japan (Supplemental Table 1).

### Ethical approval and informed consent

This study was approved by the ethics committee in each participating site (Aichi Hospital IRB approval number 2020–1-400) and all investigators complied with the Declaration of Helsinki in the conduct of the research. As this was a retrospective chart review, the collection of informed consent was not required based on the ethical guidelines for medical and health research involving human subjects in Japan.

### Information collected in the medical chart review

All information was pseudonymized and collected as part of the chart review on patient characteristics, such as age at study participation, age at first breast cancer diagnosis, weight (kgs), height (cms), body mass index (BMI) and menopausal status. Clinical characteristics such as age at abemaciclib initiation, starting abemaciclib dose, line of therapy, patients Ki67 (as determined by their treating physician using their own clinical judgement), estrogen/progesterone receptor status, and Eastern Cooperative Oncology Group (ECOG) score at treatment initiation were collected. Data on therapies received alongside abemaciclib, treatments received prior to initiation of abemaciclib, and real-world tumor response were also collected. These data were collected around the time of study initiation. Best treatment response was assessed using RECIST (Response Evaluation Criteria in Solid Tumors) criteria when this information was available in the clinical chart, or as per local practice in the rest of cases. Healthcare resource use, including tests and imaging were also examined.

### Statistical analysis

Descriptive statistics of each of key variables were presented. Frequency and proportions for categorical variables and means and medians for continuous variables were reported together with standard measures of dispersion. Progression-free survival (PFS) was calculated as the time (in months; calculated as number of days/30.5) from the first dose with abemaciclib to the first documentation of objective tumor progression or death due to any cause, whichever occurs first. Patients without records of disease progression or death were censored at the time of last observation.

Statistical analyses were performed using SAS® 9.4. [23]

### Sources of funding

The study, the associated analyses described in this manuscript, and scientific writing support for same, were all funded by Eli Lilly and Company.

## Results

### Patient characteristics

Two hundred patients with complete information from across the 14 institutions in Japan participating in the study were included in the analysis. Median age at abemaciclib initiation was 59 years (range 28–84 years) (Table 1). The mean (Standard deviation [SD]) weight was 53 kg (10 kg), mean (SD) height was 156 cm (6 cm) and mean (SD) BMI was 22 (4). ECOG performance status at the first abemaciclib dose was 0 for 51% of patients (*n* = 102) and 1 for 34% of patients (*n* = 68). Over half of our sample were post-menopausal (*n* = 116, 58%), 49 (24.5%) were pre-menopausal, 29 (14.5%) were post-induced menopause, and the remaining six (3%) were peri-menopausal (Table 1).

**Table 1:** Patient demographic and clinical characteristics

Variable	*N*=200
Median patient age, years (range)^1^	60 (28-86)
Median patient age at 1st diagnosis, years (range)^2^	51 (23-78)
Starting abemaciclib dose, *n*(%)	
150 mg	185 (92.5%)
100 mg	15 (7.5%)
ECOG score at treatment initiation, *n* (%)^3^	
0	102 (58.3%)
1	68 (38.9%)
2	5 (2.9)
Median patient age at start of abemaciclib, years (range)	59 (28-84)
Mean patient weight, kg (SD)	53.3 (9.8)
Mean patient height, cm (SD)	156.0 (6.0)
Mean BMI, index (SD)	21.9 (3.7)
Menopausal status, *n*(%)	
Premenopausal	49 (24.5%)
Peri-menopausal	6 (3.0%)
Post-menopausal	116 (58.0%)
Post-menopausal (induced)	29 (14.5%)
Cancer stage at 1st diagnosis, *n* (%)	
Stage 0	1 (0.5%)
Stage I	35 (17.5%)
Stage II	77 (38.5%)
Stage IIIa	12 (6.0%)
Stage IIIb	3 (1.5%)
Stage Illc	10 (5.0%)
Stage IV^4^	52 (26%)
Unknown	10(5%)
Disease measurability, *n* (%)^5^	
Measurable	139 (69.5%)
Treatment received prior to advanced staging, *n* (%)^4^	
Surgery	143 (99.3%)
Radiotherapy	73 (50.7%)
Neoadjuvant therapy	29 (20.1%)
Adjuvant therapy	134 (93.1%)
Perioperative treatment, *n* (%)^6^	Yes
Chemotherapy (Perioperative BC)	85 (62.0%)
Hormone therapy (Perioperative BC)	134 (97.8%)
Targeted therapy (Perioperative BC)	2 (1.5%)
Other therapy (Perioperative BC)	3 (2.2%)

^1^At the date of last information in the patient’s clinical chart

^2^There were 22 patients with missing values in age at 1st diagnosis

^3^There were 25 patients whose ECOG score was not assessed or unknown

^4^52 patients had stage IV disease at 1st diagnosis. These patients did not have to answer the question on prior therapies

^5^Based on physician criteria

^6^Based on the patients who selected “neoadjuvant” or “adjuvant” treatment prior to their advanced staging (*n*=137)

ECOG Eastern Cooperative Oncology Group. BMI Body mass index. BC Breast cancer

The most common stage when patients were initially diagnosed with breast cancer was Stage II (*n* = 77, 38.5%) followed by stage IV (*n* = 52, 26%) and Stage I (*n* = 35, 17.5%). Stage IIIa-c diagnosis was present in 25 patients (12.5%) while stage was unknown for 10 patients (5%). Measurable disease, as assessed by physician criteria, was present in 139 patients (69.5%). The predominant histological cancer type at diagnosis was invasive ductal carcinoma (*n* = 121, 61.4%). All but one patient (*n* = 199, 99.5%) was at Stage IV on receipt of their first dose of abemaciclib.

In the 148 patients for whom information of treatment received prior to advanced staging was available, 143 (99.3%) had undergone surgery, 73 (50.7%) had received radiotherapy, 29 (20.1%) received neoadjuvant therapy and 134 (93.1%) some form of adjuvant therapy. In the 137 patients who received either adjuvant or neoadjuvant treatment, 85 received chemotherapy (62.0%), 134 (94.8%) received hormone therapy, 135 (98.6%) received targeted therapies and 3 (2.2%) patients received some other form of (neo)adjuvant therapy.

### Ki67, estrogen receptor and progesterone receptor status

In total, information on biopsies from metastatic sites was available for 97 participants. Of these, 45 participants (46.4%) were found to have high Ki67 status while 17 (17.5%) had low Ki67. A further 35 (36.1%) were not tested. With respect to estrogen receptor (ER) status, 84 patients (86.6%) tested positive, six tested negative (6.2%) and seven (7.2%) were not tested. For progesterone (PR) status, 67 patients tested positive (69.1%), 21 (21.7%) tested negative, and a further 9 (9.3%) were not tested.

### Line of therapy, combination drugs and dose

**Fig. 1:**
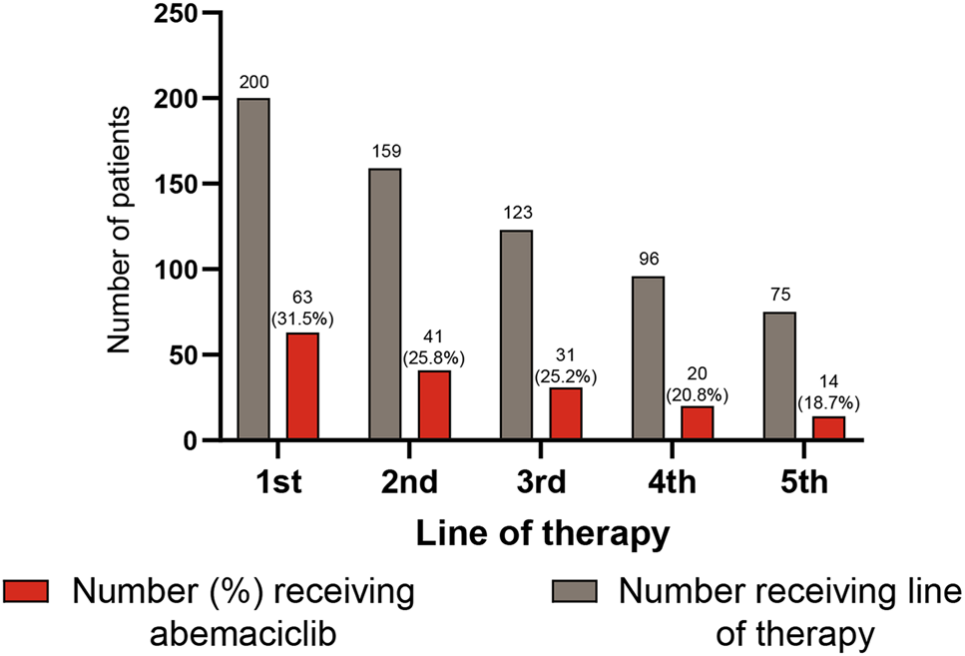
Abemaciclib receipt by line of therapy. The percentage of patients receiving abemaciclib in a particular line is based on the overall number of patients who received treatment in that line

Out of the 200 patients included in our analysis, 63 received abemaciclib as their first line of therapy for MBC. This represented 31.5% of patients in the total population. The corresponding numbers for the 2nd, 3rd, 4th and 5th line therapy were 41 (25.8%), 31 (25.2%), 20 (20.8%), and 14 (18.7%) (Fig. 1). Across all treatment lines,^1^ the most common backbone therapy with abemaciclib was fulvestrant (*n* = 116, 58.8%) (Table 2). Abemaciclib in combination with aromatase inhibitors (such as anastrozole, exemestane or letrozole) was observed in 79 patients (40.1%). When broken down by treatment line, 35 patients received abemaciclib alongside fulvestrant and 27 received abemaciclib in combination with aromatase inhibitors as the first-line therapy. For the 2nd line and 3rd and later lines, this was 31 and 10, and 50 and 42, respectively. The vast majority of the patients started abemaciclib at a dose of 150 mg (*n* = 185, 92.5%) while 15 (7.5%) received a starting dose of 100 mg. Half of the patients (51.8%) had their doses decreased while on abemaciclib therapy, with no differences according to the first dose. The patients with reduced starting dose of 100 mg were older (median 69.5 years) compared to those with standard starting dose of 150 mg (59.6 years).

**Table 2:** Combination endocrine therapies received with abemaciclib overall and by treatment line

Combination (*n*=197)^1^	Overall *n*(%)	1st line (*n*)	2nd line (*n*)	3rd line or later (*n*)
Abemaciclib + AI^2^	79 (40.1%)	27	10	42
Abemaciclib + fulvestrant	116 (59.2%)	35	31	50
Abemaciclib + other	2 (1%)	1	0	1

^1^Three patients received abemaciclib later than the 10th line. Their results are not included in this analysis

^2^Anastrozole, exemestane or letrozole

AI aromatase inhibitors

### Treatment response and survival

Tumor response information was available for 171 patients. It is to be noted that only 119 patients had their response evaluated using the RECIST criteria. Of the 171, 7 (4.1%) showed complete response, 45 (26.3%) showed a partial response, 78 (45.6%) had stable disease while the remaining 41(24%) had progressive disease (Table 3). For the remaining 29 participants, tumor response was unknown, not assessed, or was too early to ascertain. The proportion of patients who discontinued their abemaciclib treatment during the study period was 64.5% (35.5% were censored).

**Table 3:** Best treatment response

Best overall treatment response (*n*=173)^1^	*n* (%)
Complete response	7(4.1%)
Partial response	45(26.3%)
Stable disease	78(45.6%)
Progressive disease	41(24%)

^1^For 29 patients, response was unknown, not assessed, or was too early to ascertain. They were excluded from the analysis

**Fig. 2:**
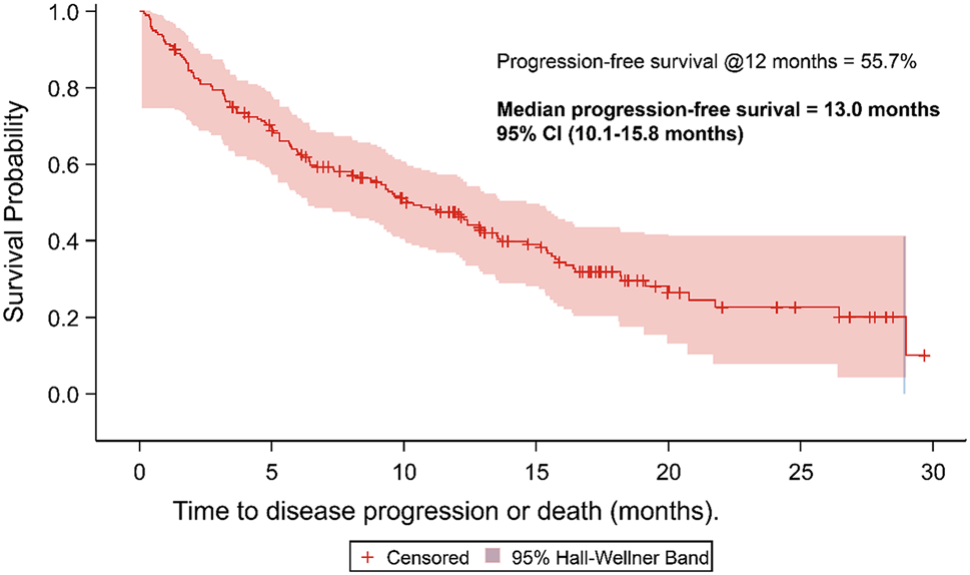
Progression-free survival

**Fig. 3:**
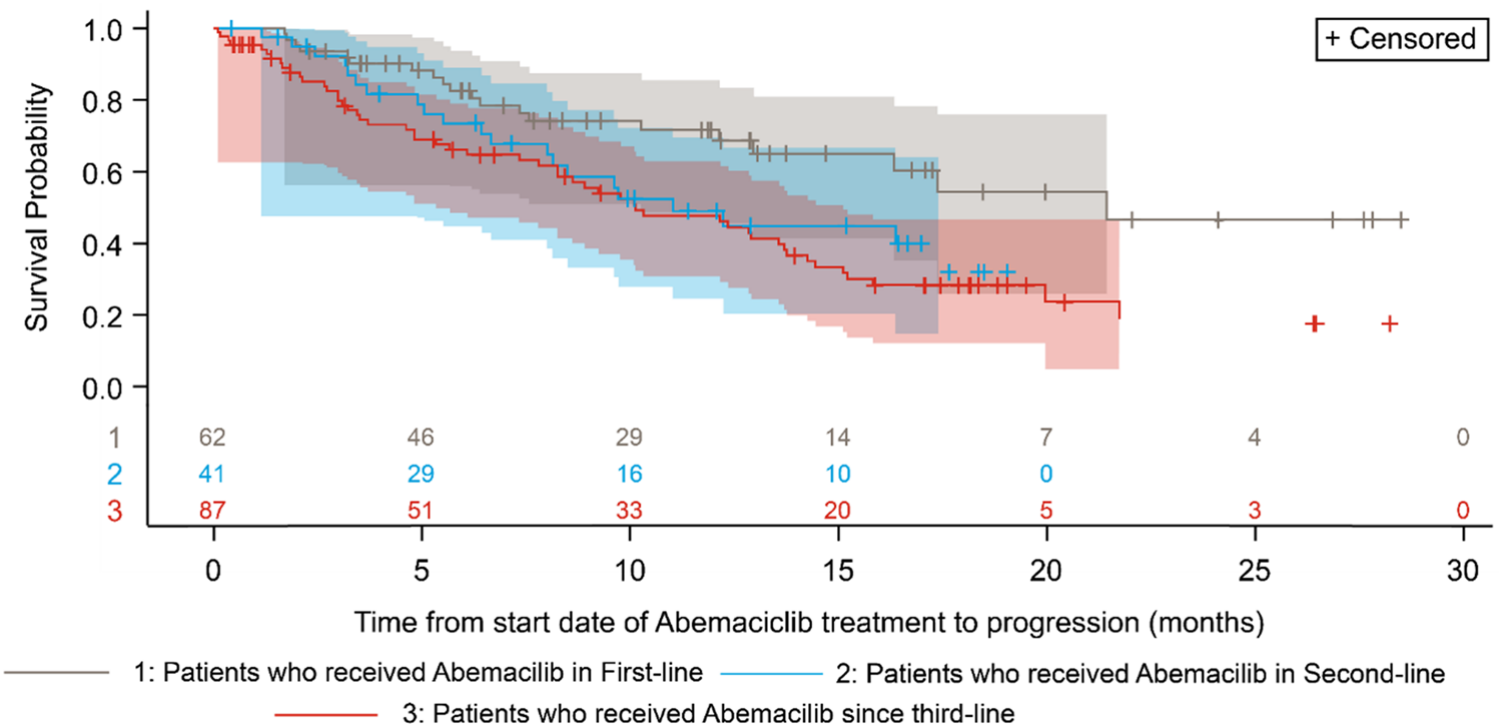
Progression-free survival by line of therapy

The median time to abemaciclib discontinuation was 308 days/10.1 months (95% confidence interval: 245–393 days/8–12.9 months) and the 1-year probability of continuing abemaciclib treatment was 47.5%. Those patients with a higher initial dose had longer median time to discontinuation (366 days vs 125 days / 12 months vs 4 months). PFS at 12 months was 55.7% while median progression-free survival was 13 months (95% CI 10.1–15.8 months) (Fig. 2). When broken down by line of treatment (Fig. 3), the proportion of patients with PFS at 12 months who received abemaciclib in 1st line was 71.6%, 2nd 49.0%, and 3rd line or later 47.7%. Median PFS in patients who received abemaciclib in 1st line, 2nd line, and 3rd line or later was 21.4 months, 11.0 months, and 10.1 months, respectively.

### Pre and post abemaciclib treatment

**Fig. 4:**
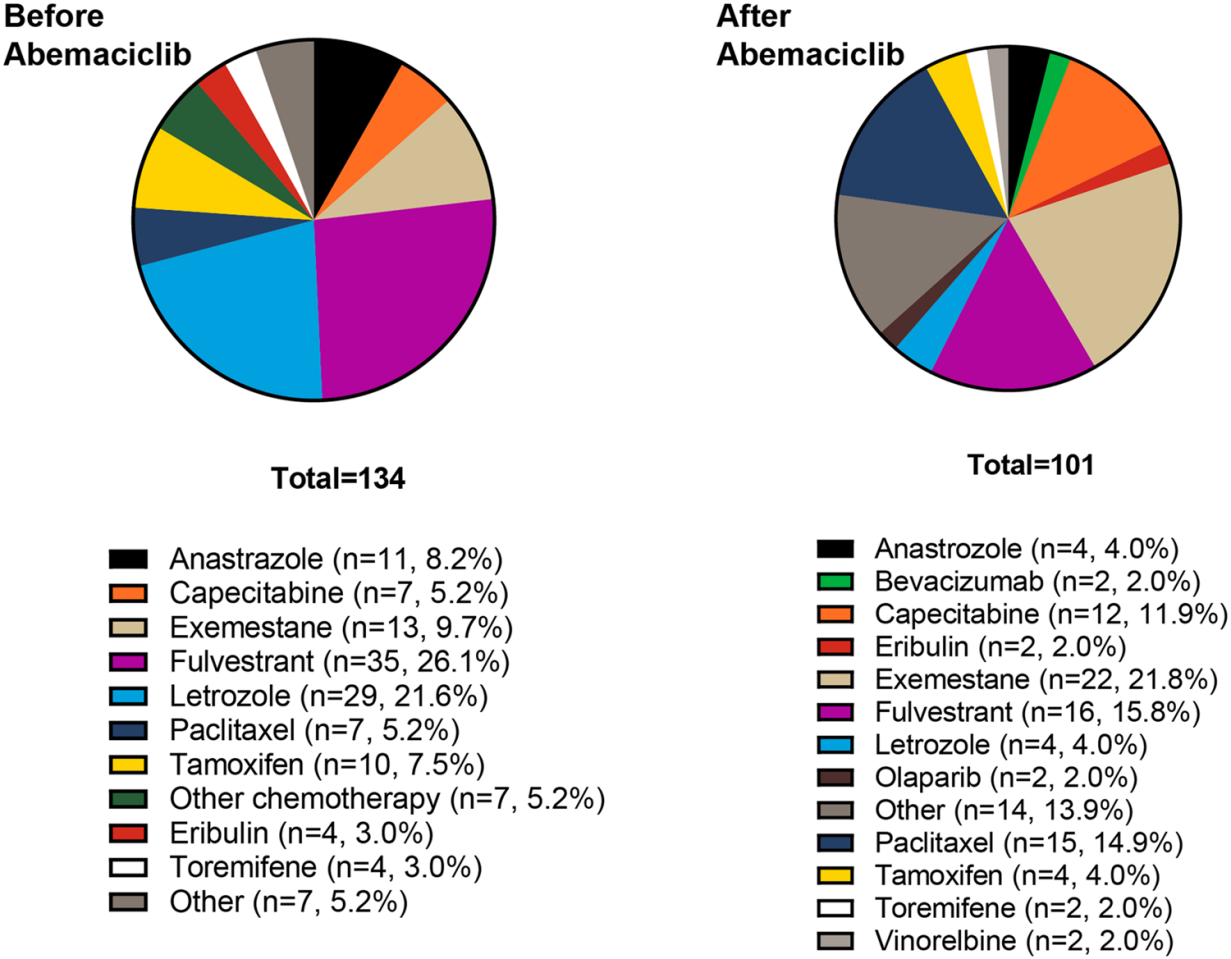
Treatments received pre and post abemaciclib initiation

The most common treatment before initiation of abemaciclib (in those who received pre-abemaciclib treatment, *n* = 134) was fulvestrant (*n* = 35, 26.1%) followed closely by letrozole (*n* = 29, 21.6%) (Fig. 4). Other common treatments were exemestane (*n* = 13, 9.7%) and anastrozole (*n* = 11, 8.2%). For those who received subsequent line of therapy after their abemaciclib treatment (*n* = 101), the most common treatment was exemestane (*n* = 22, 21.8%). This was followed by fulvestrant (*n* = 16, 15.8%), paclitaxel (*n* = 15, 14.9%), and capecitabine (*n* = 12, 11.9%).

### Healthcare resource use

Almost all patients had none (*n* = 169, 84.50%) or only a single (*n* = 23, 11.50%) hospitalization, including those for surgery, in the 3 months before their initial abemaciclib dose. This stayed the same for the three months period after abemaciclib initiation, with 168 (84.0%) of the patients did not experience hospitalizations. All patients had received some test or scan in the 3 months before their initial abemaciclib dose, the most common among which were CT scans (*n* = 154, 77%) or complete blood counts (*n* = 200, 100%).

## Discussion

Our study described and highlighted the patient characteristics, treatment patterns and outcomes of abemaciclib in women with HR + , HER2- metastatic breast cancer in routine clinical practice in Japan. In terms of patient profile and treatment characteristics, the vast majority of the patients in our sample were prescribed 150 mg as their initial dose and started abemaciclib in combination with an endocrine therapy—mostly fulvestrant. This is consistent with the label of abemaciclib in Japan. The proportion of patients who reduced abemaciclib dose (51.8%),in our real-world study, was comparable to that in the Japanese population in the RCTs; 54.0% in MONARCH2 [19], 55.3% in MONARCH3 [20]. Also of note was that while median PFS was highest when abemaciclib was used as part of first-line therapy, the drug performed very nearly as well in the second and even third lines in terms of median PFS (Fig. 3). This observation is in alignment with the latest Japanese guidelines [11] that strongly recommends CDK4/6 inhibitor in combination with ET as the first-line or second-line treatment for HR + /HER2- MBC, while the potential benefit from abemaciclib could also provide an opportunity to manage their disease in later lines.

Almost one third of patients in this cohort experienced tumor response (PR, 26%; or CR, 4%), and about a half of the patients experienced stable disease (46%). These results indicate clinical benefit, as was observed in MONARCH2 (overall response rate 35.2%) and MONARCH3 (59%) trials. [17, 24]. To correctly interpret our results, differences in assessments of effectiveness between a clinical trial and routine clinical practice should be noted. Clinical trials usually apply RECIST [25] to determine treatment response; however, as documented in the patient chart, real-world treatment response might be based on the treating clinician’s assessment of disease burden change, and not every case is defined as per RECIST criteria, which may be influenced by heterogeneous interpretation of radiological reports, and/or missing data. Such differences could bias estimates of treatment effectiveness in either direction in routine clinical practice. Another aspect that should be considered is that real-world utilization of new drugs often shows that the early post-approval period is often characterized by heterogeneity in use [26, 27], as demonstrated by varying abemaciclib use across lines of therapy (as shown in the Fig. 1, a certain number of patients were treated with abemaciclib after 3L) and regimens in this real-world cohort. In addition, the patients in our study were in worse health status in terms of ECOG PS (0 in 58.3% of patients), compared to the Japanese study population from the RCTs of abemaciclib where the ECOG PS was 0 in 93.8% (MONARCH2) [19] and 78.9% (MONARCH3) [20]. This is expected for real-world studies in general, where the patient population is much more heterogeneous compared to the strictly selected study population in clinical trials.

Despite these differences and heterogeneity, it should be acknowledged that the 12-month real-world PFS probability of 55.7% is close to the real-world PFS in a recent US study looking at abemaciclib in routine care (61.7%) [28] and the RECIST determined 12-month PFS rates seen in MONARCH 2 (61.1%) [28]. Levels of hospitalization remained low, and relatively constant, in our sample both in the period immediately before the initiation of abemaciclib and in the period after. Consequently, we saw no signs for our group of patients that the initiation of abemaciclib lead to any significant need for inpatient care and further underlines the safety profile of the drug outlined in the clinical trial evidence.

Our study represents the first large-scale consideration of abemaciclib for the treatment of HR + , HER2- MBC patients in routine clinical practice outside of the United States and provides a useful real word evidence compliment to the existing body of clinical trial data that supports the use of abemaciclib in this patient group. Compared to the only other real-world evidence reported so far in Japan [29] (a number of other studies examine safety alone [30–32]), our study has a considerably larger sample size and has wider coverage with participating sites from across Japan.

Some potential limitations should be considered when interpreting these results. Of note is that this is a report based on data from 14 participating sites from across Japan,, which may limit the potential generalizability of these results. However, our work could still represent the largest-scale real-world data source on abemaciclib for MBC in Japan so far. Another is that this study is based on a retrospective review of clinical charts. The information that can be obtained is only that recorded in patients’ clinical chart. While information regarding treatment patterns is likely to have good cross-site comparability, information relating to disease progression may be less uniformly defined, and in terms of tumor response, not all patients were evaluated using RECIST criteria. Additionally, selection of sites within the study was not random, so while the sample was geographically comprehensive, it may not be fully representative of patients receiving abemaciclib in all settings in Japan. Finally, our sample included patients who started treatment with abemaciclib during a period of nearly three years. Since this period includes the first months after abemaciclib availability in Japan, it is possible that treatment patterns may have changed during the period of data collection.

Our study evaluated real-world abemaciclib outcomes and utilization among patients with HR + , HER2- MBC, within the first 3 years following initial approval in Japan. It represents an important step in terms of understanding the patient profile and the routine care outcomes observed in patients with HR + /HER2- MBC treated with abemaciclib. Further evidence, however, is needed particularly as regards overall survival data and performance in routine clinical practice in other countries that would demonstrate greater generalizability of the findings in the current study.

## Conclusions

Our real-world study highlighted the profile, treatment pattern and outcome of patients receiving abemaciclib for metastatic breast cancer in Japan. Median PFS was comparable to the findings from clinical trials, and numerically higher in the 1st line setting and similar between the 2nd and 3rd line settings. Our study supports that, in a routine clinical practice setting, patients with HR + , HER2- MBC appeared to benefit in terms of treatment response and PFS, as in the clinical trials, from treatment with abemaciclib.

## Supplementary Information

Below is the link to the electronic supplementary material.

Supplementary file1 (DOCX 14 KB)

## Data Availability

The data that support the findings of this study are available on request from the corresponding author. The data are not publicly available due to that their containing information that could compromise the privacy of research participants.

## Footnotes

1 Information was not available for 3 patients who received abemaciclib after the 10th line.
