# Supplementary material for: Real-world treatment patterns and outcomes of abemaciclib for the treatment of HR + , HER2- metastatic breast cancer patients in Japan
Source: Breast Cancer. 2023 May 22;30(4):657–65. doi: 10.1007/s12282-023-01461-6 (PMC10284953; doi:10.1007/s12282-023-01461-6)
Supplement: Supplementary file 1 — Supplementary file1 (DOCX 14 KB) [file 12282_2023_1461_MOESM1_ESM.docx]

Supplemental Table 1: Sites included in research programme

| ***Site*** | ***n (%)*** |
| --- | --- |
| Aichi Cancer Centre Hospital | 26 (13%) |
| The Cancer Institute Hospital of Japanese Foundation for Cancer Research | 25 (12.5%) |
| National Cancer Centre Hospital | 20 (10%) |
| Akita University | 1 (0.5%) |
| Kyushu University Hospital at Beppu | 2 (1%) |
| Tokyo Metropolitan Komagome Hospital | 22 (11%) |
| Osaka City General Hospital | 3 (1.5) |
| Showa University | 20 (10%) |
| National Cancer Centre Hospital East | 20 (10%) |
| Nagoya City University | 22 (11%) |
| International University of Health and Welfare Hospital | 1 (0.5%) |
| Kindai University | 20 (10%) |
| Hyogo Cancer Centre | 14 (7%) |
| Gifu University | 4 (2%) |
| *TOTAL* | *200 (100%)* |
